# Prestin in Human Perilymph, Cerebrospinal Fluid, and Blood as a Biomarker for Hearing Loss

**DOI:** 10.1002/ohn.895

**Published:** 2024-07-11

**Authors:** Anselm Joseph Gadenstaetter, Paul Emmerich Krumpoeck, Alice Barbara Auinger, Erdem Yildiz, Aldine Tu, Christian Matula, Christoph Arnoldner, Lukas David Landegger

**Affiliations:** ^1^ Christian Doppler Laboratory for Inner Ear Research, Department of Otorhinolaryngology–Head and Neck Surgery, Vienna General Hospital Medical University of Vienna Vienna Austria; ^2^ Department of Otorhinolaryngology–Head and Neck Surgery, Vienna General Hospital Medical University of Vienna Vienna Austria; ^3^ Department of Neurosurgery, Vienna General Hospital Medical University of Vienna Vienna Austria; ^4^ Present address: Lukas David Landegger, Department of Otolaryngology–Head and Neck Surgery Stanford University School of Medicine Palo Alto CA USA

**Keywords:** biomarker, prestin, sensorineural hearing loss, vestibular schwannoma

## Abstract

**Objective:**

Determining the concentration of prestin in human blood, cerebrospinal fluid (CSF), and perilymph (PL), and evaluating its suitability as a clinical biomarker for sensori‐neural hearing loss (SNHL).

**Study Design:**

Human blood, CSF, and PL samples were intraoperatively collected from 42 patients with tumors of the internal auditory canal or with intracochlear tumors undergoing translabyrinthine or middle fossa tumor removal. Prestin concentration was measured using enzyme‐linked immunosorbent assay and linear regression analyses were performed to investigate its associations with audiological as well as vestibular test results.

**Setting:**

Tertiary referral center.

**Results:**

The median prestin concentration in blood samples of the 42 study participants (26 women, mean ± standard deviation age, 52.7 ± 12.5 years) was 1.32 (interquartile range, IQR, 0.71‐1.99) ng/mL. CSF prestin levels were significantly higher with 4.73 (IQR, 2.45‐14.03) ng/mL (*P* = .005). With 84.74 (IQR, 38.95‐122.00) ng/mL, PL prestin concentration was significantly higher compared to blood (*P* = .01) and CSF (*P* = .03) levels. Linear regression analyses showed significant associations of CSF prestin concentration with preoperative hearing levels (pure‐tone average and word recognition; *P* = .008, *R* ^2^ = 0.1894; *P* = .03, *R* ^2^ = 0.1857), but no correlations with blood or PL levels.

**Conclusion and Relevance:**

This study's findings highlight the volatile nature of prestin levels and provide the first insights into this potential biomarker's concentrations in body fluids apart from blood. Future investigations should comprehensively assess human prestin levels with different etiologies of SNHL, prestin's natural homeostasis and systemic circulation, and its temporal dynamics after cochlear trauma. Finally, clinically approved detection kits for prestin are urgently required prior to considering a potential translational implementation of this diagnostic technique.

Approximately 1.5 billion people worldwide suffer from hearing loss (HL), of which 430 million cases range from moderate to complete HL and require rehabilitation.^1^, ^2^ Impairment of hearing often places severe physical, psychological, and socioeconomic stress on patients and is a known risk factor for many other diseases, such as depression and dementia.^3^ The overall cost of unaddressed HL worldwide is estimated to be above $980 billion per year, underlining the economic sequelae of the condition.^1^

Current diagnosis of HL relies on audiometric testing and is aided by screening methods, such as self‐assessment questionnaires, physical examination, imaging techniques, and different in‐office hearing tests.^4^, ^5^ As these procedures typically require expensive special equipment, time, and trained personnel, there is an urgent need for new diagnostic tools, which are quick, safe, and effective at diagnosing HL. To this end, there has been extensive research aimed at finding suitable biomarkers, potentially able to also predict the future hearing outcome. Because over 90% of HL cases are of sensorineural origin (as opposed to conductive HL),^6^ and because the pathology is located inside the cochlea in almost 90% of sensorineural HL (SNHL) cases, this research has been directed toward finding molecules that are specific to the inner ear.^7^, ^8^ Of many different identified proteins, microRNAs, and cytokines, one of the most promising candidates is the outer hair cell (OHC) protein prestin.^7^, ^8^

Prestin is encoded by the *SLC26A5* gene and although earlier reports had suggested that it was also present in vestibular hair cells,^9^ it is exclusively produced in cochlear OHCs.^8^, ^10^ Here, it is situated in the cells' lateral wall and acts as the motor protein enabling their electromotility and therefore their function of amplifying basilar membrane motion and regulating cochlear sensitivity.^10^, ^11^, ^12^ Thus, prestin deficiency causes loss of OHC electromotility and is associated with HL of approximately 40 to 60 dB.^13^, ^14^

Due to its size of roughly 80 kDa, it is assumed that the protein can cross the blood‐labyrinth barrier (BLB), and prestin has been previously detected in human and animal blood.^8^, ^11^ Here, it is possible to measure minute quantities of prestin through enzyme‐linked immunosorbent assay (ELISA), possibly in a state of OHC loss of less than 1%, which is long before cochlear damage manifests itself in audiometric testing.^11^ Multiple studies have investigated prestin levels in animals and humans with different etiologies of SNHL (eg, idiopathic, noise‐induced, or cisplatin‐induced) and proposed prestin as both a diagnostic and a prognostic biomarker for SNHL.^11^, ^15^, ^16^, ^17^, ^18^

To the best of our knowledge, previous investigations of prestin levels have been limited to the bloodstream, although it can be assumed that in case of OHC damage, prestin is primarily released directly into perilymph (PL) and subsequently cerebrospinal fluid (CSF). However, the possibilities of obtaining specimens from these compartments are severely limited due to their inaccessibility and the possible damage caused by the sampling procedures.^19^, ^20^ While novel techniques could establish PL sampling as a stand‐alone intervention in the future,^21^, ^22^ current methods are only safe when applied in conjunction with cochlear implantation or vestibular schwannoma (VS) resection.^20^, ^23^, ^24^, ^25^

Consequently, it is unknown whether prestin can be found in PL or CSF of patients with and without SNHL. The aim of this study was to provide a first insight into prestin levels in these compartments and to investigate if PL and CSF levels show any correlation to blood levels or to clinical parameters such as the severity of HL.

## Methods

This study was conducted in conformity with the Declaration of Helsinki and was approved by the ethics committee of the Medical University of Vienna (EK No. 1164/2022).

### Study Design and Data Acquisition

This study was performed at a single tertiary referral center in Vienna, Austria. Blood, CSF, and PL samples of patients with tumors originating from the internal auditory canal (IAC) or intracochlear tumors were collected prospectively from May 1, 2020, to March 31, 2023, via the Austrian Network for Neuro‐Oncology tumor bank (EK No. 1375/2018). Only samples from patients who underwent either a middle fossa (MF) or translabyrinthine surgical approach for tumor removal were collected for this study after obtaining written informed consent. No cases with retrosigmoid approach were included as this technique does not involve otolaryngologists at the study center. PL was sampled from the lateral semicircular canal with sterile disposable aspirators (VTL‐14301/VTL‐14314; Vitrolife) during labyrinthectomy and CSF was withdrawn when opening the meninges. Furthermore, whole blood samples were obtained in a 9 mL CAT Serum Clot Activator tube (Vacuette®; Greiner) during tumor removal (“intra” blood samples), allowed to clot for 20 to 30 minutes at room temperature, and then centrifuged at 2000*g* for 10 minutes, and serum was stored for subsequent analysis. Additionally, in a subset of patients, more blood samples were obtained prior to surgery (“pre”) as well as directly after wound closure (“post”; ergo, at the end of surgery) and processed as described above. All specimens were immediately transferred to a safe lock tube and stored at −80°C until prestin concentration was determined using ELISA. This was performed with a human prestin (*SLC26A5*) ELISA kit (MBS8808028; MyBioSource) per the manufacturers' protocol. Each well's optical density was measured at 450 nm using a microplate reader (Tecan Spark®; Tecan Group), and data were collated using SparkControl™ software (Version 2.2). Hemolyzed blood samples, and CSF or PL samples with blood contamination were excluded from further analysis.

Clinical data were collected from the in‐house patient database and included patient demographics, information on the surgery, radiological information on tumor size and location (Koos^26^ and Hannover^27^ grading systems), tumor histology, most recent pre‐, and if available postoperative audiometric measurements, and latest preoperative vestibular assessment prior to surgery. Data from pure‐tone audiometry are presented as the pure‐tone average of 4 frequencies (4‐PTA; 0.5/1/2/4 kHz). Word recognition score (WRS) was evaluated using the German Freiburg monosyllabic word test^28^ at 80 dB and is presented as the percentage of correctly recognized words. For vestibular assessment, results from video head impulse and caloric reflex tests (Otosuite®, Otometrics; Natus Medical Inc) are presented as directional preponderance and unilateral weakness, ranging from 100% ipsilateral (+100) to 100% contralateral (−100).

### Statistical Analysis

Clinical data are reported individually and are presented as either counts and proportions for categorical variables, mean ± standard deviation for normally distributed, or median with the interquartile range (IQR) for not normally distributed variables. For comparisons between 2 groups, a Mann‐Whitney *U* test was used as data were not normally distributed; for calculations involving more than 2 groups, a univariate Welch's analysis of variance (ANOVA) with Dunnett's T3 post hoc test was performed. For comparisons between prestin concentrations in pre‐, intra‐, and postoperative blood samples, a repeated measures ANOVA was performed applying the Greenhouse‐Geisser adjustment if correction for violations of sphericity was required. Correlation was evaluated using Spearman's correlation coefficient (*r*_s_). For investigation of the relationship between prestin concentration and clinical parameters, linear regression analyses were performed. For all comparative analyses, a 2‐sided *P* < .05 was considered statistically significant, and in case of multiple comparison correction, the adjusted *P* value (*P*_adj_) is given.

Statistical analyses were performed using IBM SPSS (Version 29; IBM). GraphPad Prism (Version 9.1.0; GraphPad) was used for linear regression analyses, and to compile graphs.

## Results

### Study Population

**Figure 1:**
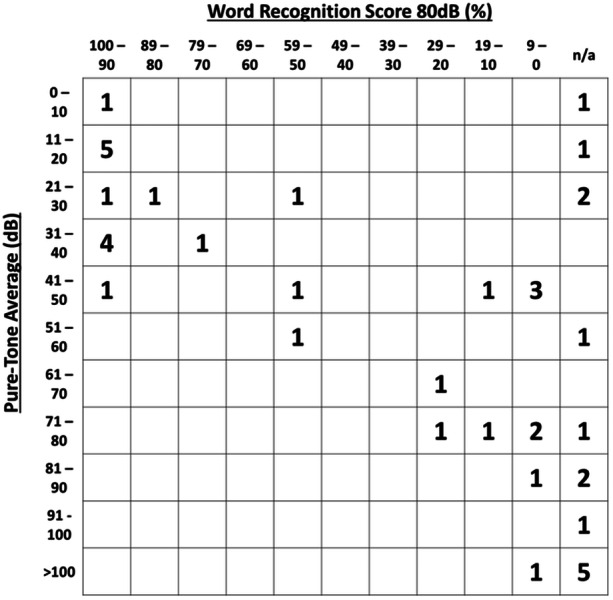
Scattergram of preoperative audiometric results. Pure‐tone averages are represented on the *y*‐axis and word recognition scores at 80 dB on the *x*‐axis. The numbers represent patients whose audiometric data place them into certain squares.

Samples were obtained from 42 different patients undergoing IAC or intracochlear tumor removal (Table 1). The mean age at the time of surgery was 52.7 ± 12.5 years. Of the 26 women and the 16 men included in this analysis, a majority suffered from VS (32/42; 76.2%) and were operated on using a translabyrinthine approach (24/42; 57.1%). Of note, 2 of the included individuals with VS had undergone preoperative gamma knife radiosurgery, with one being affected by neurofibromatosis type II‐associated VS. Preoperatively, the patients' median 4‐PTA HL was 48.8 (IQR, 25.6‐78.4) dB with 10 patients having normal hearing (4‐PTA ≤ 25 dB), whereas the median WRS at 80 dB was 62.5 (IQR, 2.5‐95)% (n = 28; Figure 1). Overall, no significant differences in 4‐PTA of air and bone conduction were observed in the patients. With a median 4‐PTA of 12.5 (IQR, 7.5‐32.5) dB, contralateral 4‐PTA hearing levels were significantly lower compared to ipsilateral hearing (*P* < .001). Subdividing the preoperative ipsilateral PTA into low (0.125/0.25/0.5 kHz), speech (1/2/3 kHz), and high (4/6/8 kHz) frequencies showed significantly higher PTA in high frequencies compared to low frequencies [59.17 (IQR, 44.17‐93.33 dB) vs 33.33 (IQR, 16.25‐73.33 dB), *P* = .02], yet proved indifferent to speech‐frequency PTA [46.67 (IQR, 28.33‐86.67 dB), *P* = .82]. A similar result was observed on the contralateral ear with high‐frequency PTA being significantly higher than low‐frequency PTAs [25.00 (IQR, 14.17‐46.67 dB)] vs 11.67 (IQR, 7.50‐18.33 dB), *P* = .0232], but not compared to speech‐PTA [11.67 (IQR, 5.00‐30.83 dB), *P* = .70]. Of note, however, was that no associations between age and neither ipsilateral 4‐PTA (*R* ^2^ = 0.0003, *P* = .92) and high‐frequency PTA (*R* ^2^ = 0.0156, *P* = .43) nor contralateral 4‐PTA (*R* ^2^ = 0.0043, *P* = .68) and high‐frequency PTA (*R* ^2^ = 0.0343, *P* = .25) were found.

**Table 1:** Descriptive Characteristics of Included Patients

Characteristic	Patient status (n = 42)
Sex, No. (%)	
Female	26 (61.9)
Male	16 (38.1)
Age at surgery, y	
Mean ± SD [range]	52.7 ± 12.5 [22–81]
Median (IQR)	54 (43‐62)
Tumor histology, No. (%)	
Vestibular schwannoma	32 (76.2)
Hemangioma	5 (11.9)
Meningioma	2 (4.8)
Other	3 (7.1)
Tumor laterality, No. (%)	
Left	22 (52.4)
Right	20 (47.6)
Tumor size for vestibular schwannoma, No. (%)	(n = 32)
Koos grading	
Grade 1	23 (71.9)
Grade 2	5 (15.6)
Grade 3	1 (3.1)
n/a (intracochlear schwannoma)	3 (9.4)
Hannover grading	
Grade 1	23 (71.9)
Grade 2	4 (12.5)
Grade 3	1 (3.1)
Grade 4	1 (3.1)
n/a (intracochlear schwannoma)	3 (9.4)
Surgical approach, No. (%)	
Translabyrinthine	24 (57.1)
Middle fossa	18 (42.9)
Preoperative pure‐tone hearing threshold, No. (%)	
Mean ± SD	54.4 ± 33.4
Median (IQR)	48.8 (25.6‐78.4)
No hearing loss; 4‐PTA ≤ 25 dB	10 (23.8)
Mild hearing loss; 4‐PTA 26‐40 dB	8 (19.0)
Moderate hearing loss; 4‐PTA 41‐60 dB	8 (19.0)
Severe hearing loss; 4‐PTA 61‐80 dB	6 (14.3)
Profound hearing loss; 4‐PTA ≥ 81 dB	10 (23.8)
Preoperative word recognition score (at 80 dB)	(n = 28)
Mean ± SD	54.3 ± 42.5
Median (IQR)	62.5 (2.5‐95)

Abbreviations: 4‐PTA, pure tone average of 4 frequency; dB, decibel; IQR, interquartile range from 25% to 75% quartile; n/a, not available; SD, standard deviation; y, years.

Intraoperative blood samples were available for all but 1 patient (97.6%), CSF samples for a total of 36 patients (85.7%), and PL samples for 12 patients (28.6%). Matching pre‐, intra‐, and postoperative blood samples were available for a total of 11 patients (26.2%). The exact compilation of available samples can be found in Supplemental Table S1, available online.

### Comparison of Prestin Concentration in Different Body Fluids

**Figure 2:**
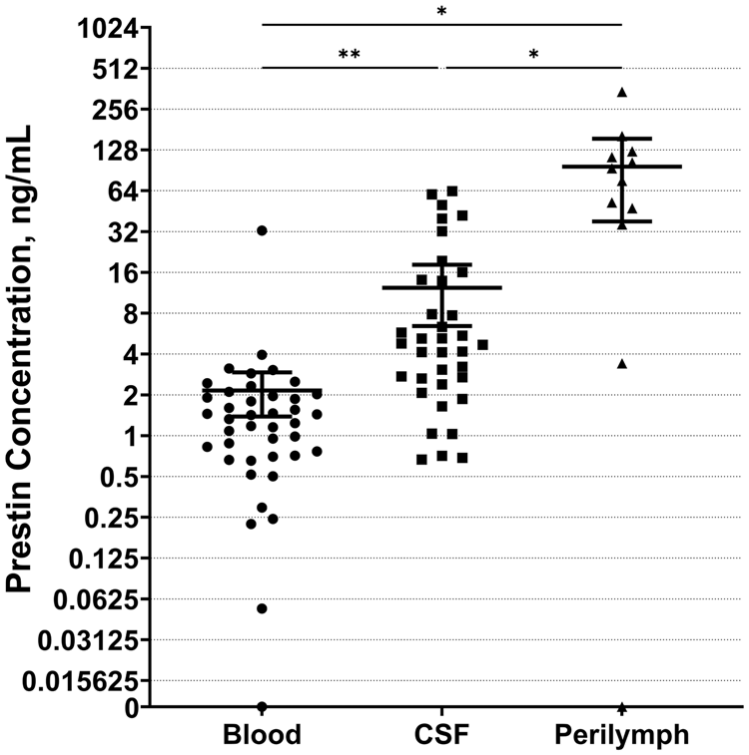
Comparison between prestin concentrations in intraoperative blood, cerebrospinal fluid, and perilymph samples. Data are presented as means ± standard error of the mean. Asterisks indicate significant differences (*P < .05; **P < .01). CSF, cerebrospinal fluid.

The median prestin concentration in intraoperative blood samples was 1.32 (IQR, 0.71‐1.99) ng/mL. In CSF samples, the median prestin concentration was 4.73 (IQR, 2.45‐14.03) ng/mL. In PL samples, the median concentration was 84.74 (IQR, 38.95‐122.00) ng/mL. Overall, the prestin concentration was significantly higher in PL compared to intraoperative blood and CSF samples (*P*_adj_ = .01, 95% CI = 20.32‐167.99; *P*_adj_ = .03, 95% CI = 10.01‐157.98), and higher in CSF compared to intraoperative blood (*P*_adj_ = .005, 95% CI = 2.70‐17.61; Figure 2). However, no correlation between any of the 3 concentrations was observed (blood‐CSF: *r*_s_ = −0.045, *P* = .80; blood‐PL: *r*_s_ = −0.112, *P* = .73; CSF‐PL: *r*_s_ = −0.191, *P* = .57).

The median prestin concentration in preoperative blood samples was 0.59 (IQR, 0.51‐1.49) ng/mL, whereas the median concentration in postoperative blood samples was 0.86 (IQR, 0.31‐1.39) ng/mL. No statistically significant difference in prestin concentration between pre‐, intra‐, and postoperative blood samples was detected (*P* = .22; Supplemental Figure S1, available online). The same was observed when subgrouping patients according to the exerted surgical approach (MF: *P* = .55; translabyrinthine: *P* = .19). Prestin blood concentration levels were positively correlated between all 3 (pre‐intra‐post) timepoints (pre‐intra: *r*_s_ = 0.690, *P* = .05; intra‐post: *r*_s_ = 0.709, *P* = .02; pre‐post: *r*_s_ = 0.755, *P* = .007).

No significant differences in intraoperative blood, CSF, and PL prestin levels were observed between male and female patients (*P* = .81; *P* = .15; *P* = .73; Supplemental Figure S2a, available online) or in patients with VS or other IAC tumors (*P* = .90; *P* = .96; *P* = .50; Supplemental Figure S2b, available online). Moreover, no differences in blood or CSF levels were found in patients undergoing an MF versus translabyrinthine approach (*P* = .26; *P* = .27; Supplemental Figure S2c, available online).

### Association of Prestin Concentration and Clinical Parameters

Linear regression analyses were performed to investigate the relationship between prestin concentration of intraoperative blood, CSF, and PL with preoperative 4‐PTA, WRS, and vestibular parameters (directional preponderance and unilateral weakness). For analyses with intraoperative blood levels, 1 outlier was excluded (32.56 ng/mL). As no associations between prestin levels and age at surgery (blood: *R* ^2^ = 0.0003, *P* = .91; CSF: *R* ^2^ = 0.0069, *P* = .63; and PL: *R* ^2^ = 0.1127, *P* = .29) were found, no corrections for age were performed.

**Figure 3:**
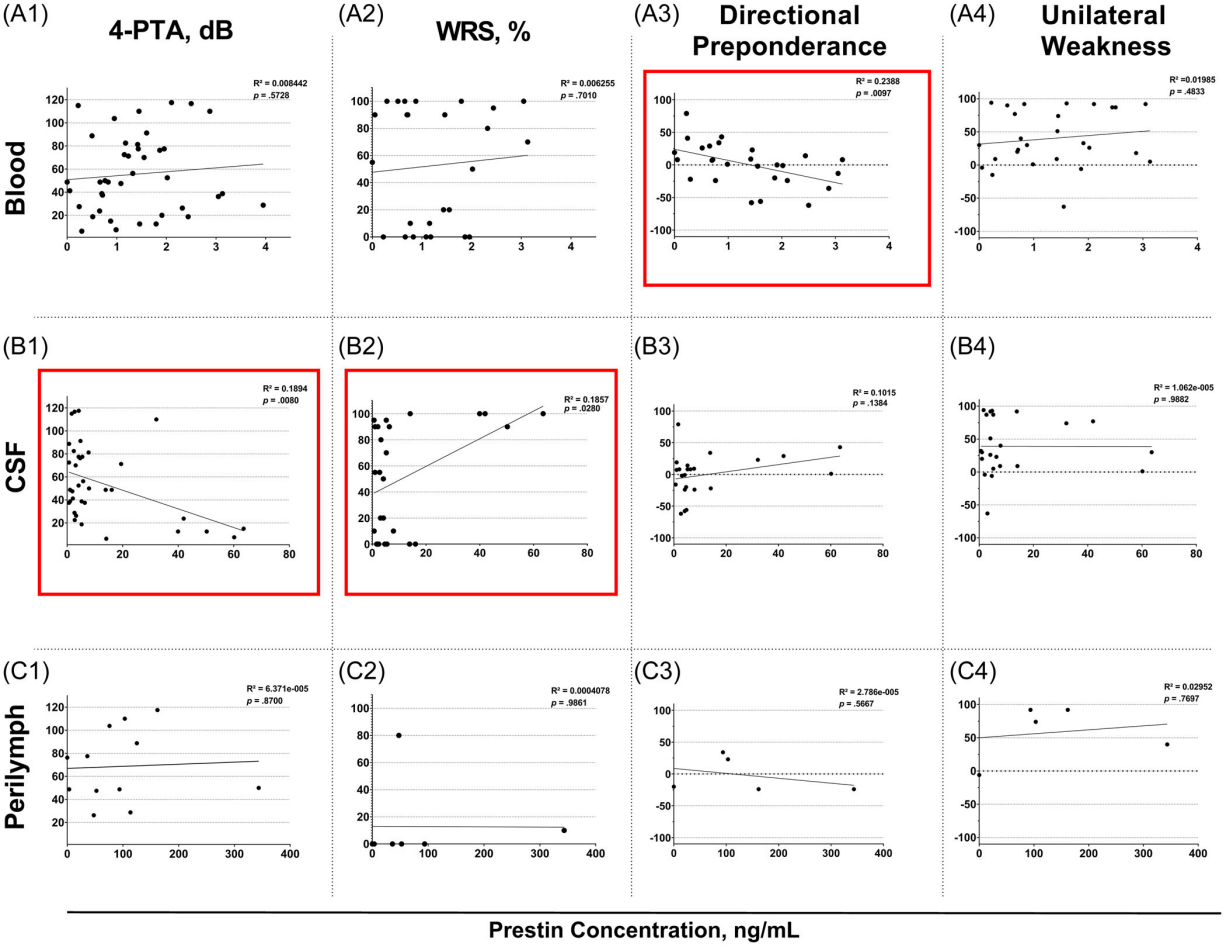
Linear regression analyses of prestin concentrations and preoperative clinical parameters. Relationship between prestin in blood (A1–A4), CSF (B1–B4), and perilymph (C1–C4) with preoperative 4‐PTA (A1–C1), WRS (A2–C2), directional preponderance (A3–C3), and unilateral weakness (A4–C4). Red boxes indicate significant associations. 4‐PTA, pure‐tone average; CSF, cerebrospinal fluid; WRS, word recognition score.

Three out of all the possible associations produced significant results (Figure 3). Blood prestin level significantly corresponded to the preoperative directional preponderance (*R* ^2^ = 0.2388; *P* = .01), and prestin concentration in CSF was significantly associated with preoperative 4‐PTA (*R* ^2^ = 0.1894; *P* = .008) as well as WRS (*R* ^2^ = 0.1857; *P* = .03). Subdividing the preoperative PTA of all tested frequencies into low, speech, high, or all tested frequencies revealed that CSF prestin levels were significantly associated with speech (*R* ^2^ = 0.2033; *P* = .006), and high (*R* ^2^ = 0.2686; *P* = .002) frequencies (Supplemental Figure S3, available online). Moreover, no statistically significant associations between the contralateral 4‐PTA and prestin levels in any of the 3 fluid compartments were observed (blood: *R* ^2^ = 0.0157, *P* = .45; CSF: *R* ^2^ = 0.0910, *P* = .08; and PL: *R* ^2^ = 0.0458, *P* = .50).

Finally, the possible predictive value of prestin levels in intraoperative blood and CSF samples on postoperative hearing preservation in patients after MF surgery and the hearing outcome in patients after translabyrinthine surgery with concomitant cochlear implantation was evaluated (Supplemental Figure S4, available online). However, no associations between prestin concentration in blood and CSF samples with changes in 4‐PTA (preoperative 4‐PTA minus postoperative 4‐PTA) were observed in patients 3.8 ± 6.0 months after MF surgery (*R* ^2^ = 0.0160, *P* = .67; *R* ^2^ = 0.1211, *P* = .29). Likewise, no significant correlations between prestin levels in blood and CSF with postoperative 4‐PTA after an average of 7.4 ± 2.8 months were detected in patients after translabyrinthine surgery and subsequent cochlear implantation (*R* ^2^ = 0.1591, *P* = .25; *R* ^2^ = 0.1790, *P* = .30).

## Discussion

Early diagnosis of HL remains critically important for providing appropriate care and support.^29^ Even though the use of hearing aids cannot slow the clinical progression of HL, it can immensely impact patients' everyday lives, benefitting speech perception,^30^ communication,^31^ and overall quality of life.^32^ Furthermore, early fitting with hearing aids could decrease the risk of cognitive impairments associated with HL.^33^, ^34^, ^35^ Today, as stated above, appropriate biomarkers are lacking. An optimal biomarker would represent a cost‐effective, reliable, and expeditious alternative to conventional means to diagnose HL along with potentially allowing an objectification and classification of the degree of HL.

Multiple different biomarkers have been investigated for numerous otologic diseases in patients, for example, matrix metalloprotease 14 for VS,^36^, ^37^ OTOLIN‐1 for SNHL and Ménière's disease,^38^ or various plasma metabolites for tinnitus.^39^ Besides those, prestin also was proposed and investigated as a suitable biomarker to diagnose SNHL in previous studies. As such, blood prestin levels were found to be elevated in patients with SNHL,^40^, ^41^ and idiopathic sudden SNHL.^18^, ^42^ In contrast, a reduction of prestin levels was observed after cisplatin exposure^43^ or in patients with age‐related HL.^44^ Furthermore, blood prestin levels were elevated in patients with noise‐induced HL,^45^ yet also reduced in normal‐hearing subjects with increased noise exposure.^46^ While the exact mechanisms of prestin release into the systemic circulation are still poorly understood, there are a few hypotheses, which could explain these somewhat contradictory findings. Increased prestin levels may result from direct release of prestin after OHC damage into the systemic circulation followed by (temporary) upregulation of prestin expression in the remaining OHCs, whereas a decrease in prestin levels after cochlear damage could stem from a disequilibrium of cochlear homeostasis with the remaining OHCs releasing less prestin into the systemic circulation.^8^, ^46^

Nevertheless, all previous studies investigating prestin as a potential biomarker for SNHL have only evaluated its protein level in samples obtained from peripheral blood. Hitherto, our study is the first to also determine prestin concentrations in CSF and PL. While intracellular proteins of organ of Corti cells have been detected within the PL following cochlear trauma, the exact passage into the PL remains unclear.^47^ In the present study, we found that prestin concentrations significantly differ between the 3 distinct sample liquids as concentrations were highest in PL followed by CSF and lowest in blood samples. Of particular interest, however, was the fact that the concentrations between the 3 fluids did not correlate with each other. This suggests that the concentration of systemically circulating prestin does not actually mirror the concentration within the cochlea itself. We hypothesize that not only prestin concentration in the PL alone, but also the permeability of the BLB may influence the systemic distribution of prestin. It has long been suggested that various diseases eliciting SNHL, including VS, can damage the BLB and increase its permeability.^48^ Furthermore, we observed that surgical destruction of the vestibular system—as is the case in the translabyrinthine approach—did not significantly elevate blood prestin levels directly after trauma (Supplemental Figure S1, available online). While this could be explained by the immediate removal of drilled tissue via suction as it occurs during labyrinthectomy, it could be seen as another proof of absence of prestin in vestibular hair cells as in other studies, changes of blood prestin levels occurred as early as 4 hours (to 1 day) after the initial trauma and should therefore also have been detected in our analysis.^16^, ^49^, ^50^ Considering the previously reported temporal shifts of prestin concentration after cochlear trauma or therapy,^16^, ^42^, ^49^, ^50^ this leads us to believe that differences of prestin concentration may also be explained by various temporal factors, for example, time since disease onset or deterioration. Even though it was believed that prestin is a protein uniquely expressed in cochlear OHCs, there has been a single report of its presence outside of the inner ear, namely in cardiomyocytes.^51^ While these results still lack external validation, this would undoubtedly challenge prestin's suitability as a biomarker for SNHL.

Surprisingly, the previously reported relation between blood prestin concentration and degree of HL^17^, ^40^, ^45^ could not be observed in our study. We speculate that patients even with subclinical or mild HL in our cohort could already present with elevated blood prestin concentrations and devoid of direct comparison to a healthy patient cohort without otologic disease, these elevated prestin levels might not be visible. We would expect part of this patient subcategory to have some minor degree of cochlear damage as VSs have been observed to secrete factors damaging inner ear structures.^52^, ^53^ Nevertheless, previous studies have also shown that HL observed in conventional audiograms up to 8 kHz is accurately correlated to actual hair cell loss found in postmortem human specimens proving this method's practicability in predicting cellular damage.^54^ However, instead of a correlation of clinically detectable HL with blood prestin levels, we demonstrated a negative association of CSF's prestin concentration with 4‐PTA together with a positive association with WRS indicating that higher prestin levels in CSF corresponded to better preoperative hearing. This might be explained by the aforementioned dysregulation of cochlear homeostasis in severely damaged cochleae leading to less prestin being exuded into the CSF.^8^, ^46^ The significant association between CSF prestin levels with speech‐ and high‐frequency PTA could be explained by an interplay between incipient age‐related HL as seen in elevated high‐frequency PTA on the contralateral, healthy ear and tumor‐induced damage predominantly affecting the basal cochlear region.

### Strengths and Limitations

Our study represents the first analysis to investigate prestin levels not only in blood samples, but also in human CSF and PL specimens. By demonstrating differences of the prestin concentration in these 3 compartments, we shed more light on the exact mechanisms regulating prestin distribution in body fluids. Further preclinical and translational studies will have to be carried out in order to gain a deeper understanding of physiological prestin circulation within the cochlear fluids and systemic blood system, and its potential suitability as a clinical biomarker for HL.

Due to the aforementioned difficulties associated with sample collection of human PL, our study suffers from a small and therefore heterogenous patient cohort mainly differing in tumor entities observed, age at surgery, and clinical degree of HL. Furthermore, the lack of some samples in few subjects additionally diminished comparability, notably of PL concentrations. Missing samples were a consequence of exclusion of hemolyzed or imperfect specimens (eg, bloody CSF) and seldom failure to collect samples intraoperatively. The resulting lack of power may contribute to the absence of statistical significance in few of the performed tests and could prompt further investigations in these particular scenarios. Lastly, including a normal‐hearing control group would have certainly increased the validity of this study's results; but, as previously discussed, current PL sampling techniques can be highly deleterious to the cochlea and typically cause permanent damage to the inner ear, making the enrollment of healthy subjects unacceptable.

Compared to other previously published studies, we found great differences in reported mean blood prestin concentrations. This could be explained by the different etiologies of SNHL as in our case, retrocochlear pathology elicited by the tumors may have induced HL in the absence of cochlear (hair cell) damage associated with changes of prestin level. However, we believe that the selection of different ELISA kits may influence final prestin concentrations to a greater extent as all currently available products are for research use only. For our study, we selected a kit with a higher detection range due to the anticipated higher CSF and PL concentrations and found blood concentrations comparable to Sun et al^42^ With a reported intra‐assay precision of <8% (coefficient of variation) and interassay precision of <10% (coefficient of variation), we paid special attention to analyzing all individual patients’ samples on the same ELISA plate. Studies using kits with lower detection ranges, however, reported concentrations, which were roughly 10‐fold lower compared to the findings of this study, something which cannot solely be explained by the reported intra‐assay variation.^18^, ^40^, ^45^

## Conclusions

To date, our study is the first to investigate prestin levels in other body fluids besides blood, namely in CSF and PL. We show that prestin levels greatly differ between these 3 compartments. Although, we detected novel associations between levels of HL and prestin in CSF, the previously reported associations between blood prestin concentration and hearing levels were not observed in our study. Future studies should assess and correlate prestin levels in blood and PL in other forms of SNHL, for example, in cochlear implant recipients or various preclinical animal models. Nevertheless, there is still an unmet need for clinically approved prestin detection kits, which are urgently required to improve comparability of results obtained by different studies and laboratories.

## Author Contributions

**Anselm Joseph Gadenstaetter**, concept and design, acquisition, analysis, or interpretation of data, drafting of the manuscript, statistical analysis; **Paul Emmerich Krumpoeck**, concept and design, acquisition, analysis, or interpretation of data, drafting of the manuscript; **Alice Barbara Auinger**, acquisition, analysis, or interpretation of data, critical revision of the manuscript for important intellectual content; **Erdem Yildiz**, acquisition, analysis, or interpretation of data, critical revision of the manuscript for important intellectual content; **Aldine Tu**, acquisition, analysis, or interpretation of data, critical revision of the manuscript for important intellectual content; **Christian Matula**, acquisition, analysis, or interpretation of data, critical revision of the manuscript for important intellectual content; **Christoph Arnoldner**, acquisition, analysis, or interpretation of data, critical revision of the manuscript for important intellectual content, obtained funding; **Lukas David Landegger**, concept and design, acquisition, analysis, or interpretation of data, critical revision of the manuscript for important intellectual content, obtained funding, supervision.

## Disclosures

### Competing interests

Prof Christoph Arnoldner has received research funding from MED‐EL and Audiocure. Dr Lukas David Landegger has received research funding from Decibel Therapeutics/Regeneron Pharmaceuticals and Amgen and has worked as an independent consultant for Conclave Capital and Gerson Lehrman Group. No other disclosures were reported.

### Funding source

Dr Lukas David Landegger is supported by a Career Development Award from the American Society of Gene & Cell Therapy and the Children's Tumor Foundation. The content is solely the responsibility of the authors and does not necessarily represent the official views of the American Society of Gene & Cell Therapy. The financial support of the Austrian Federal Ministry for Digital and Economic Affairs, the National Foundation for Research, Technology and Development, and the Christian Doppler Research Association is gratefully acknowledged.

## Supporting information

Supplementary Figure 1. Comparison of Prestin Concentrations in Pre‐, Intra‐, and Postoperative Blood Samples. Connected data points indicate samples from individual patients. Abbreviations: ns, not significant.

Supplementary Figure 2. Differences of Prestin Concentrations. Prestin concentrations of intraoperative blood, CSF, and perilymph samples grouped according to a) sex, b) tumor histology, and c) surgical approach.

Supplementary Figure 3. Linear Regression Analyses of Prestin Concentrations and Preoperative PTA Divided into Low, Speech, High, and All Frequencies. Red boxes indicate significant associations between prestin concentration and clinical parameters.

Supplementary Figure 4. Linear Regression Analyses of Prestin Concentrations and Postoperative Clinical Parameters. Relation of blood and CSF prestin levels with pure‐tone threshold shift after middle‐fossa surgery or PTA after translabyrinthine surgery with cochlear implantation.

Supporting information.
