# Supplementary figures and images for: Prestin in Human Perilymph, Cerebrospinal Fluid, and Blood as a Biomarker for Hearing Loss
Source: Otolaryngol Head Neck Surg. 2024 Jul 11;171(6):1825–33. doi: 10.1002/ohn.895 (PMC11605028; doi:10.1002/ohn.895)

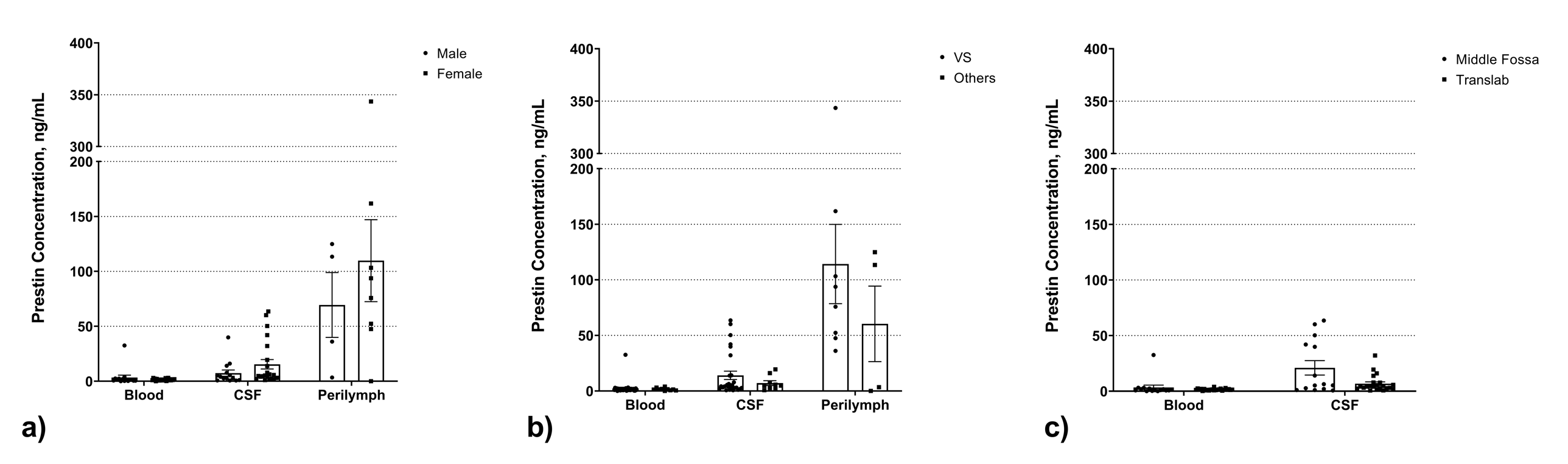

Supplement: Supplementary file 2 — Supplementary Figure 2. Differences of Prestin Concentrations. Prestin concentrations of intraoperative blood, CSF, and perilymph samples grouped according to a) sex, b) tumor histology, and c) surgical approach. [file OHN-171-1825-s003.tif]
