# Supplementary material for: Prestin in Human Perilymph, Cerebrospinal Fluid, and Blood as a Biomarker for Hearing Loss
Source: Otolaryngol Head Neck Surg. 2024 Jul 11;171(6):1825–33. doi: 10.1002/ohn.895 (PMC11605028; doi:10.1002/ohn.895)
Supplement: Supplementary file 5 — Supporting information. [file OHN-171-1825-s002.docx]

**Supplementary Material**

| **Available Samples** | **No. (%)** |
| --- | --- |
| Intra Blood only | 5 (11.9) |
| Intra Blood + CSF | 20 (47.6) |
| Intra Blood + CSF + PL | 5 (11.9) |
| Pre+Intra+Post Blood + CSF | 4 (9.5) |
| Pre+Intra+Post Blood + CSF + PL | 6 (14.3) |
| Pre+Intra+Post Blood + PL | 1 (2.4) |
| Pre Blood + CSF | 1 (2.4) |

Supplementary Table 1. Compilation of Available Samples

Abbreviations: CSF, cerebrospinal fluid; PL, perilymph; Pre, preoperative; Intra, intraoperative; Post, postoperative
